# ﻿*Synodusautumnus*, a new species of lizardfish (Aulopiformes, Synodontidae) from the Indo-Pacific region, and a reassessment of distributional records of *Synodusrubromarmoratus*

**DOI:** 10.3897/zookeys.1243.147259

**Published:** 2025-06-26

**Authors:** Ryusei Furuhashi, Hiroyuki Motomura

**Affiliations:** 1 The United Graduate School of Agricultural Sciences, Kagoshima University, 1-21-24 Korimoto, Kagoshima 890-0065, Japan Kagoshima University Kagoshima Japan; 2 The Kagoshima University Museum, 1-21-30 Korimoto, Kagoshima 890-0065, Japan The Kagoshima University Museum Kagoshima Japan

**Keywords:** Description, morphology, *Synodusbinotatus*, *Synoduslobeli*, taxonomy, Teleostei

## Abstract

The Indo-Pacific lizardfish *Synodusautumnus***sp. nov.** (Aulopiformes, Synodontidae) is morphologically distinct from all other nominal species of *Synodus*, and is described as new. It is characterized by the following combination of characters: dorsal-fin rays 11–13; anal-fin rays 8–10; lateral-line scales 49–51; scale rows above lateral line 3.5; scale rows below lateral line 4.5; vertebrae 49–52; anterior gill rakers 22–29; peritoneal spots 0–5; anterior palatine teeth in a discrete group, longer than posterior palatine teeth; anterior nostril flap long, broad, leaf-like, extending above and behind posterior margin of posterior nostril when laid back; posterior process of pelvic girdle wide; posterior part of preopercle scaled; body with 5 reddish saddle-like blotches; and lateral surface below lateral line with a straight row of brown blotches when fresh. The new species is similar to *Synodusbinotatus* Schultz, 1953 and *Synodusrubromarmoratus* Russell & Cressey, 1979, but these species differ in having 52–56 and 53–55 lateral-line scales, respectively. In addition, *S.binotatus* has blotches below the lateral line in a zigzag pattern, and *S.rubromarmoratus* has anterior and posterior palatine teeth similar in length, 14–25 teeth on the tongue, and 0–5 peritoneal spots. No color pattern differences were apparent between the new species and *S.rubromarmoratus*, making differentiation between the two species in underwater photographs impossible; consequently, underwater photographs and unsupported catalog records were excluded from the reassessment of each species’ distribution. Examination of specimens reported as *S.rubromarmoratus* revealed that the true *S.rubromarmoratus* has been collected only in Australian waters, whereas *S.autumnus***sp. nov.** is widely distributed in the eastern Indian and Pacific oceans.

## ﻿Introduction

*Synodusrubromarmoratus* was described by Russell and Cressey (1979) based on specimens obtained from the east coast of Australia, but has since been recorded extensively from the Indo-Pacific region (Russell and Cressey 1979; Cressey 1981; Randall et al. 1990; Anderson et al. 1998; Randall 1998, 2005, 2007; Sadovy and Cornish 2000; Hutchins 2001; Kuiter and Tonozuka 2001; Allen and Adrim 2003; Myers and Donaldson 2003; Heemstra et al. 2006; Kuiter and Debelius 2006; Senou et al. 2006a, 2006b, 2007; Fricke et al. 2011; Allen and Erdmann 2012; Russell 2022; Gloerfelt-Tarp and Kailola 2022). Many records of *S.rubromarmoratus* have been based on underwater photographs (e.g., Randall et al. 1990; Anderson et al. 1998; Randall 1998, 2005; Sadovy and Cornish 2000; Senou et al. 2006a, 2006b, 2007; Allen and Erdmann 2012), especially because the red blotches have been considered a unique and diagnostic feature of the species. In addition, the species is distinguished from all other valid species of *Synodus* as follows: 54 or 55 lateral-line scales, 3.5 scale rows above lateral line, 11 or 12 peritoneal spots, posterior part of preopercle scaled, anterior and posterior palatine teeth same length, anterior nostril flaps long and broad, and body with reddish saddle-like blotches (Russell and Cressey 1979; Cressey 1981; Waples and Randall 1988; Russell 1999, 2002; Chen et al. 2007; Prokofiev 2008; Randall and Pyle 2008; Randall 2009; Ho et al. 2010, 2016; Frable et al. 2013; Allen et al. 2017; Polanco and Acero 2021; Furuhashi and Motomura 2025).

However, detailed examination of Pacific Ocean specimens of *Synodus* with coloration similar to the above revealed a hitherto unknown species, differing in some morphological aspects from the type specimens of *S.rubromarmoratus*. The former is herein described as a new species, and comparisons are made with *S.rubromarmoratus*.

## ﻿Material and methods

Counts and measurements followed Furuhashi and Motomura (2025). Measurements were made to the nearest 0.1 mm with calipers. Distance from pelvic-fin origin to dorsal fin origin abbreviated as P–D line. Osteological characters were observed from soft X-ray photographs. Curatorial procedures for KAUM specimens followed Motomura and Ishikawa (2013). The holotype of *S.autumnus* sp. nov. is deposited at KAUM (Kagoshima University Museum, Kagoshima University, Kagoshima, Japan), and other specimens examined in this study are deposited in the following museums and universities: AMS (Australian Museum, Sydney, Australia), ASIZP (Biodiversity Research Museum, Chinese Academy of Sciences, Taipei, Taiwan), BPBM (Bernice Pauahi Bishop Museum, Honolulu, USA), KAUM, KPM (Kanagawa Prefectural Museum of Natural History, Odawara, Japan), NSMT (National Museum of Nature and Science, Tsukuba, Japan), NTM (Museums and Art Galleries of the Northern Territory, Darwin, Australia), USNM (Museum Support Center, Smithsonian Institution, National Museum of Natural History, Suitland, USA).

For DNA barcoding, total DNA was extracted from muscle tissue preserved in 99.5% ethanol, using the Wizard Genomic DNA Purification Kit (Promega Inc.), according to the manufacturer’s protocol. The partial cytochrome *c* oxidase subunit I (COI) gene was amplified using the primers designed by Ward et al. (2005) (Fish F1: 5'-TCAACCAACCACAAAGACATTGGCAC-3' and Fish R1: 5'-TAGACTTCTGGGTGGCCAAAGAATCA-3'). PCR was conducted in a 25 µL reaction volume containing Go Taq Green Master Mix (Promega Inc.) 7.5 µL, 0.25 µM each of forward and reverse primers, and 1.0 µM of template DNA. The thermal regime consisted of an initial step for 3 min at 94 °C, followed by 30 cycles comprising 94 °C for 30 s denaturation, 46 °C for 30 s annealing, and 65 °C for 40 s extension, with a final extension for 10 min at 65 °C. The PCR products were visualized on 1.2% agarose gels. Sequencing of the samples was performed at Dragon Genomics Center, Takara Bio Inc., Otsu, Japan. The following two specimens of *Synodusautumnus* sp. nov. were sequenced in this study (KAUM–I. 82280: LC859380; KAUM–I. 180000: LC859381), the sequence data being deposited at the International Nucleotide Sequence Database Collaboration (INSDC: https://www.insdc.org/) via the DNA Data Bank of Japan (DDBJ). The sequences determined here were aligned using Clustal W (Thompson et al. 1994), along with data of *Synodus* already published by INSDC (accession numbers: MK657570, MK658125, MK658445, MK658662, *S.binotatus*; OQ386840, OQ387399, OQ387293, PP966353, *S.autumnus* sp. nov.; MF409619, S.cf.autumnus) or BOLD Systems (accession numbers: FUT135-18, FUT452-18, *S.binotatus*). From the aligned sequences (598 base pairs), a pairwise matrix of simple uncorrected distance (*p*-distance) was constructed using MEGA 11 software (Tamura et al. 2021), and the best evolutionary model found by MEGA 11 software (Tamura et al. 2021), the T92+G model being selected (Tamura 1992). A neighbor-joining phylogenetic tree was reconstructed using MEGA 11, and branch support was measured using nonparametric bootstrapping with 1000 replications, based on the same algorithm (Felsenstein 1981).

### ﻿Comparative material examined

*Synodusbinotatus*: USNM 140801, holotype, 84.3 mm SL, Kwajalein Atoll, Marshall Islands, 1 Sept. 1946, E. Herald; USNM 401320 (MK658445), 83.6 mm SL, Mangareva, Tuamotu-Gambier, French Polynesia, 8–12 m depth, 3 Oct. 2010, J. Williams, S. Planes, E. Delrieu-Trottin, M. Veuille and C. Menniti; USNM 423426 (MK657570), 107.3 mm SL, Tubuai, Austral Islands, French Polynesia, 3–5 m, 16 Apr. 2013, J. Williams, E. Delrieu-Trottin and P. Sasal; USNM424048 (MK658125), 96.6 mm SL, Rimatara, Austral Islands, French Polynesia, 20–26 m, 18 Apr. 2013, J. Williams, E. Delrieu-Trottin and P. Sasal. *Synoduslobeli* Waples & Randall, 1988: BPBM 28869, 2 paratypes, 38.4–86.8 mm SL, off Kailua, Kona Coast, Hawaiian Islands, 32 m, 18 June 1982, J. E. Randall and P. S. Lobel; BPBM 29293, holotype, 116.6 mm SL, BPBM 30337, 9 paratypes, 51.0–92.7 mm SL, off Kailua, Kona Coast, Hawaiian Islands, 32 m, 8 Aug. 1983, J. E. Randall, L. H. Strauss and C. J. Boyle; USNM 136282, 2 specimens, 67.5–70.6 mm SL, Lagonoy Gulf, the Philippines, 18 June 1909; USNM 217615, 4, 43.2–73.1 mm SL, Pingtung, Taiwan, 23 Apr. 1968, V. Springer, J. Choat, W. Boughner and K. Chang. *Synodusrubromarmoratus*: AMS I. 19450-027, holotype, 73.8 mm SL, AMS I. 19450-024, 10 paratypes, 50.6–71.2 mm SL, USNM 218792, 2 paratypes, 67.1–68.8 mm SL, Mrs Watsons Bay, Lizard Island, Queensland, Australia, 15 m, 10 Nov. 1975, B. C. Russell.

## ﻿Results

### ﻿ Synodus autumnus sp. nov.

Taxon classificationAnimaliaAulopiformesSynodontidae

440EC599-5E6A-51A0-8A9B-1262C8599C72

https://zoobank.org/06C5C528-E186-4C6E-AF6C-33834F22152B

**Figure 1.**
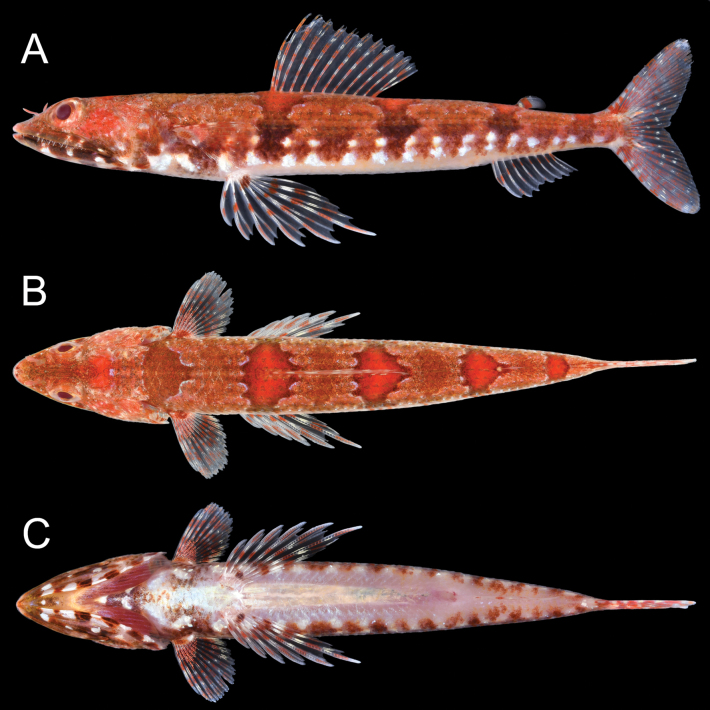
Fresh holotype of *Synodusautumnus* sp. nov. (KAUM–I. 180000, 54.2 mm SL, Segaura, Kushi, Bonotsu, Minami-satsuma, Satsuma Peninsula, southern Kyushu, Japan). **A.** Lateral view; **B.** Dorsal view; **C.** Ventral view.

**Figure 2.**
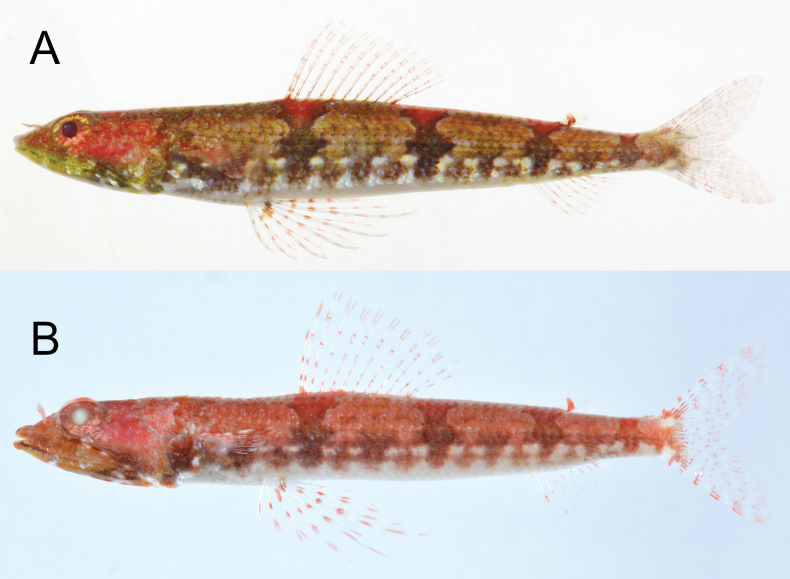
Fresh paratypes of *Synodusautumnus* sp. nov. **A.**KAUM–I. 82280, 58.3 mm SL, Japan; **B.**KPM-NI 43326, 45.8 mm SL, Japan, photo by H. Senou.

**Figure 3.**
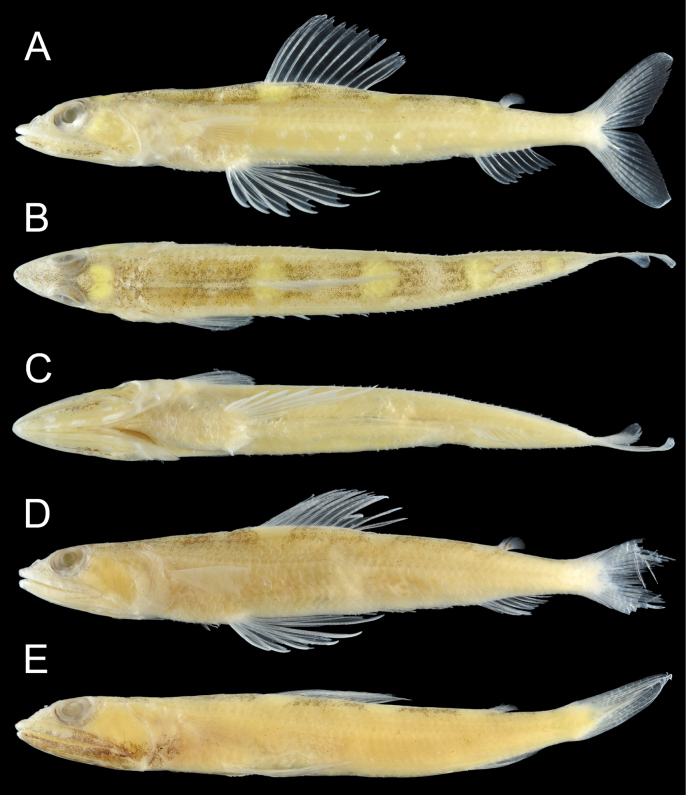
Preserved specimens of *Synodusautumnus* sp. nov. **A**–**C.**KAUM–I. 180000, holotype, 54.2 mm SL, Japan; **D.**KAUM–I. 82280, paratype, 58.3 mm SL, Japan; **E.**USNM 436037, paratype, 57.2 mm SL, the Philippines. **A, D, E.** Lateral view; **B.** Dorsal view; **C.** Ventral view.

**Figure 4.**
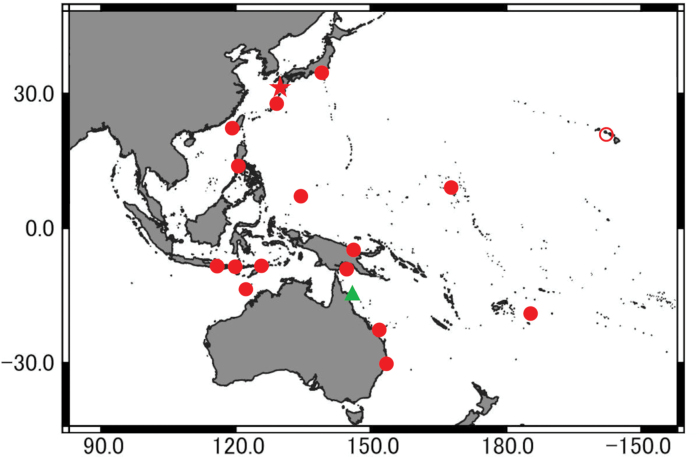
Distributional records of *Synodusautumnus* sp. nov. (red star, closed and open circles indicate holotype, paratypes and genetic sample, respectively) and *Synodusrubromarmoratus* (green triangle indicates type locality).

**Figure 5.**
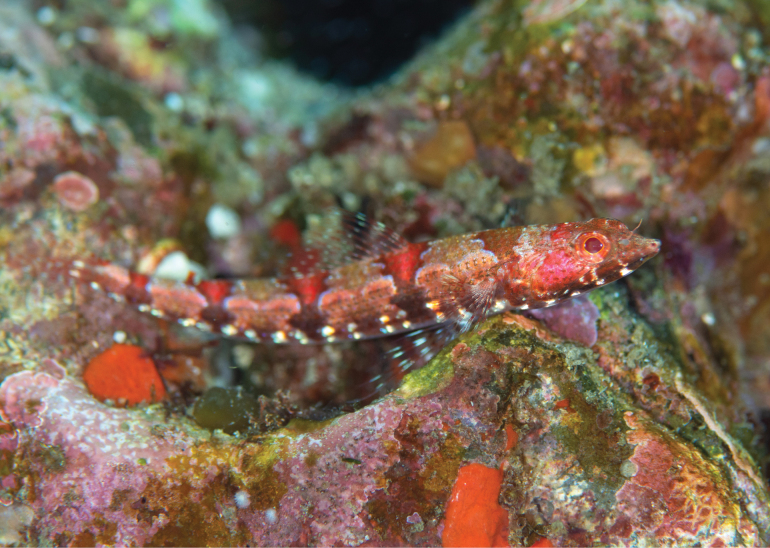
Underwater photograph of holotype of *Synodusautumnus* sp. nov. (KAUM–I. 180000, 54.2 mm SL) just before collection at a depth of 5 meters off Segaura, Satsuma Peninsula, southern Kyushu, Japan. Photo by M. C. Sato.

Figs 1 , 2 , 3 , 4 , 5 , 7 , Table 1 New English name: Autumn Lizardfish; new standard Japanese name: Iroha-eso

Synodus ulae (not of Schultz): Mochida and Motomura 2018: 8 (San, Tokuno-shima island, Amami Islands, Japan).
Synodus cf. rubromarmoratus : Honda et al. 2024: 141 (east coast of Izu Peninsula, Sagami Bay, Japan).

#### Material examined.

***Holotype*.** • KAUM–I. 180000 (LC859381), 54.2 mm SL, off Segaura, Kushi, Bonotsu, Minami-satsuma, southwest coast of Satsuma Peninsula, Kagoshima Prefecture, southern Kyushu, Japan, 5 m depth, hand net, 2 Feb. 2023, coll. by M. C. Sato. ***Paratypes*.** 34 specimens, 15.8–96.5 mm SL. **Japan**: • KAUM–I. 82280 (LC859380), 58.3 mm SL, San, Tokunoshima, Tokuno-shima island, Amami Islands, Kagoshima Prefecture, 1–18 m, hand net, 25 Nov. 2015, H. Motomura, D. Uyeno, H. Uyeno, Y. Fukui, K. Eguchi and A. Yoshiura; • KPM-NI 32554, 40.6 mm SL, Izu Oceanic Park, Jogasaki Coast, east coast of Izu Peninsula (Sagami Bay), Shizuoka Prefecture, 16 Dec. 1989, H. Masuda; • KPM-NI 43326, 45.8 mm SL, Izu Oceanic Park, Jogasaki Coast, east coast of Izu Peninsula (Sagami Bay), Shizuoka Prefecture, 27 m, 5 Jan. 2017, W. Takase. **Taiwan**: • ASIZP 59174, 96.5 mm SL, Liuqiu Island, Pingtung, 23 Apr. 1984, K.-T. Shao. **Philippines**: • BPBM 22462, 2 specimens, 48.9–50.1 mm SL, Caban Island, Batangas Province, Luzon, 30 m, 28 July 1978, J. E. Randall, G. W. Tribble, R. P. H. Rutherford and K. E. Carpenter; • USNM 436037 (OQ387399), 57.2 mm SL, off coastline south of Escarceo Point, Puerto Galera, Oriental Mindoro, 12–18 m, 2 Apr. 2015, J. Williams, D. Catania and R. Betancur; • USNM 436288 (OQ387293), 68.8 mm SL, Puerto Galera, Oriental Mindoro, 23–26 m, 16 Apr. 2015, J. Williams, D. Catania and D. Dumale. **Palau**: • BPBM 9363, 4, 15.8–59.7 mm SL, Ulebsechel Island, 15.2 m, 16 Apr. 1970, J. E. Randall, A. R. Emery et al. **Marshall Islands**: • BPBM 39704, 45.4 mm SL, Bigej-Meck Reef, Kwajalein Atoll, 25 m, 29 Dec. 2004, B. D. Greene. **Indonesia**: • BPBM 32414, 67.8 mm SL, east of Toro Liu Point, Komodo, 26–28 m, 16 Oct. 1987, J. E. Randall and E. Clark; NSMT-P 106331, 52.9 mm SL, coral reef between Meno and Air islands, Lombok, Indonesia, 10 m. **Timor-Leste**: • AMS I. 46120-005, 2, 54.4–73.4 mm SL, halfway between Hera and Metinaro, 150 m off shore, 12 m, 23 Sept. 2012, M. A. McGrouther, S. E. Reader, A. Hay, J. M. Leis, B. C. Russell and P. B. Berents. **Papua New Guinea**: • AMS I. 33751-019, 45.7 mm SL, Portlock Reef, 5–31 m, 29 Jan. 1993; NTM S. 13661-036, 3, 42.0–64.4 mm SL, Madang, 18–22 m, 5 Oct. 1992, H. K. Larson and M. Jebb. **Australia**: • AMS I. 19223-001, 3, 57.4–73.0 mm SL, One Tree Island, Queensland, 30–33 m, 14 Sept. 1974, B. C. Russell; • AMS I. 30310-030, 7, 25.7–71.0 mm SL, North Solitary Island, New South Wales, 12–14 m, 5 May 1977; NTM S. 11389-040, 44.9 mm SL, north east side of Scott Reef North, Western Australia, 7–10 m, 13 Sept. 1984, B. C. Russell. **Tonga**: • AMS I. 46738-107, 83.2 mm SL, Vaka'eitu Island, Vava’n Group, 10–19 m, 13 Jan. 2015, S. E. Reader, N. Jolly, I. Middleton and T. Trnski; • AMS I. 46739-034, 41.5 mm SL, north east of O’ua Island, Ha’apai Group, 13–16.5 m, 14 Jan. 2015, S. E. Reader, N. Jolly, I. Middleton, T. Trnski, R. Robinson and C. Bedford.

#### Diagnosis.

A new species of *Synodus* with the following combination of characters: Dorsal-fin rays 11–13; anal-fin rays 8–10; lateral-line scales 49–51; scale rows above lateral line 3.5; scale rows below lateral line 4.5; vertebrae 49–52; anterior gill rakers 22–29; peritoneal spots 0–5; anterior palatine teeth in a discrete group, longer than posterior palatine teeth; ANF long and broad, leaf-like, extending above and behind posterior margin of posterior nostril when laid back; posterior process of pelvic girdle wide; posterior part of preopercle scaled; body with 5 reddish saddle-like blotches; lateral surface below lateral line with a straight row of brown blotches when fresh.

#### Description.

Data for holotype presented first, followed by paratype data in parentheses (if different). Counts and measurements in Table 1. Characters given in diagnosis not repeated.

**Table 1.** Counts and measurements of *Synodusautumnus* sp. nov.

	Holotype	Paratypes	
	KAUM–I. 180000	*N* = 34	
Standard length (mm; SL)	54.2	15.8–96.5	
Counts			mode
Dorsal-fin rays	12	11–13	12
Anal-fin rays	9	8–10	9
Pectoral-fin rays	12	11–12	12
Pelvic-fin rays	8	8	8
Caudal-fin rays	19	19	19
Lateral-line scales	50	49–51	50
Scale rows above lateral line	3.5	3.5	3.5
Scale rows below lateral line	4.5	4.5	4.5
Pre-dorsal-fin scale rows (Prd)	15	13–17	15
Pre-adipose-fin scale rows (Pra)	14	13–16	15
Post-adipose-fin scale rows (Poa)	9	7–10	8
Total of Prd, Pra and Poa	38	35–41	38
Circumpeduncular scales	14	11–14	12
Vertebrae	50	49–52	50
Anterior gill rakers	10 + 19 = 29	7–12 + 14–18 = 22–29	11 + 15 = 26
Posterior gill rakers	2 + 11 + 0 = 13	1–3 + 7–12 + 0 = 8–15	3 + 8 + 0 = 10
Pseudobranchial filaments	14	13–21	20
Peritoneal spots	0	0–5	1
Procurrent caudal-fin rays	14 + 12 = 16	13–16 + 12–14 = 25–30	15 + 13 = 28
Measurements (% SL)			mean
Pre-dorsal-fin length	42.4	40.7–45.7	43.7
Pre-adipose-fin length	82.5	79.6–84.7	82.5
Pre-anal-fin length	78.2	77.4–81.4	79.6
Pre-pectoral-fin length	29.2	28.3–32.2	29.9
Pre-pelvic-fin length	36.4	36.9–41.3	38.3
Head length	28.6	27.9–32.9	30.2
Body depth at pelvic-fin origin	13.5	10.5–17.2	14.2
Body depth at anal-fin origin	9.8	8.9–11.2	10.4
Body width	12.7	11.1–15.6	13.8
Pelvic girdle width	6.8	6.2–7.9	7.2
Pectoral-fin length	13.7	12.1–16.3	13.8
Pelvic-fin length	25.5	23.9–29.3	26.3
Longest dorsal-fin ray length	13.7	11.0–18.3	14.4
Dorsal-fin base length	17.5	15.2–18.2	16.9
Longest anal-fin ray length	7.8	7.0–9.7	8.3
Anal-fin base length	9.4	8.7–11.0	9.9
Caudal-peduncle length	11.8	10.5–13.3	12.1
Caudal-peduncle depth	5.4	5.4–6.6	5.9
Caudal-peduncle width	4.6	3.5–5.2	4.2
Snout length	7.4	6.6–8.7	7.5
Orbit diameter	6.8	5.4–8.6	6.7
Interorbital width	2.2	1.8–3.0	2.4
Postorbital length	16.2	16.3–20.0	17.8
Upper-jaw length	19.6	18.0–23.1	19.7
ANF length	2.6	1.7–3.1	2.4
ANF width	0.7	0.5–1.2	0.9
IND	0.9	0.6–1.5	1.1
ANF length / IND	3.5	1.4–3.8	2.2

Body elongate, cylindrical, body depth greatest at pelvic-fin origin. Scales moderately large, cycloid, not deciduous, present on cheek and opercle. Cheek fully scaled, 5 (4–7) vertical scale rows, becoming progressively smaller posteriorly. Pre-dorsal-fin scales not reaching a vertical line of posterior edge of orbit. No scales on dorsal, anal, adipose, and paired fins. Caudal fin with large, pointed scales on each lobe, not reaching margin of fin fork. Procurrent caudal-fin rays without scale covering.

Snout moderately pointed in dorsal view. Two nostrils, about equal size, close to each other, located on a line connecting anteriormost margin of orbit and snout tip, close to front of orbit, anterior nostril with a dermal flap on posterior margin. Posterior nostril moderately narrow, almost directly behind anterior nostril. Internarial distance about equal to posterior nostril diameter. Eye circular, directed laterally. Interorbital region broad, with slight V-shaped concavity in front view.

Mouth large, terminal, slightly oblique, gape almost reaching posterior margin of preopercle. Teeth on both jaws numerous, small, needle-like, generally two rows on upper jaw and three rows on lower jaw, outer teeth smaller, inner teeth longer, covered by lip, tip of upper jaw teeth visible, base of jaw teeth hidden when mouth closed. Palatine teeth in 2 rows anteriorly, 2 (2 or 3) posteriorly, outer row teeth long, tooth rows close together anteriorly but slightly separated. Vomerine teeth absent. Tongue short, spatulate, fleshy, with about 33 (29–50) posteriorly depressible teeth, forming a rectangular teeth patch, 5 (5 or 6) rows on posterior region. Lower-jaw length similar to upper jaw, its anterior end fitting into groove between teeth on upper-jaw tip. Gill rakers very small, unobtrusive, plate-like, with numerous tooth-like spines.

Dorsal-fin origin just behind to midpoint between snout and adipose-fin origin, dorsal-fin base longer than anal-fin base, first and second rays unbranched, second (second or third) ray longest, posterior rays gradually shorter. Adipose fin small, above midpoint of anal-fin base. Anal fin short, posteriormost ray branched to base, others unbranched, third (second or third) ray longest, remaining rays subequal. Anus just anterior to anal-fin origin. Pectoral and pelvic fins with moderately long axillary scales at bases. Pectoral fin rounded, extending beyond P–D line, uppermost ray unbranched. Pelvic fin longer than pectoral fin, sixth ray longest, innermost (eighth) ray distinctly longer than outermost (first) ray. Caudal fin forked, lobes moderately pointed, dimensions of upper and lower lobes symmetrical.

***Color in fresh specimens*** (Figs 1, 2). Body above lateral line orangish-brown (orangish or yellowish-brown), below white. Five brown blotches along lateral line connecting to reddish saddle-like blotches edged blueish-white. Blotch above pectoral-fin base indistinct. A row of distinct brown blotches and two rows of white blotches on ventrolateral surface. Head red above maxilla, dark brown (dark brown or yellowish-green) below lower jaw. Snout tip with (with or without) a pair of dark spots. Iris orangish-red (orangish-red or yellowish-orange). All fins with reddish bars, membrane uniformly translucent.

***Color in preserved specimens*** (Fig. 3). Most fresh colors lost. Body pale, except dorsum. Dorsal surface dark, areas of saddle-like blotches (when fresh) white. Lower jaw with (with or without) black pigmentation. Snout tip with (with or without) a pair of dark spots. All fins unpigmented. Peritoneum pale.

#### Distribution and habitat.

*Synodusautumnus* sp. nov. is widely distributed in the eastern Indian and Pacific oceans, from Japan, Taiwan, the Philippines, Palau, the Marshall Islands, Indonesia, Timor-Leste, Papua New Guinea, Australia, Tonga, and the Hawaiian Islands (Fig. 4). It inhabits inshore areas with coral or rocky reefs and boulders, at depths of 1–33 m (Fig. 5).

#### Etymology.

Scientific and English names of the new species are derived from its body color, which is reminiscent of shrub and tree colors that change in autumn. “Iroha” means the leaves of plants that change color in autumn.

#### Remarks.

The peritoneal spots of *Synodus* are not usually lost in species of *Synodus*, but in *S.autumnus* and *S.binotatus*, they peel off easily, making accurate counting difficult. Although this characteristic is limited to the latter two species, the peritoneal spots may be lost in examples of other species if the abdomen has decayed (Furuhashi unpubl. data).

## ﻿Discussion

### ﻿Comparisons

**Figure 6.**
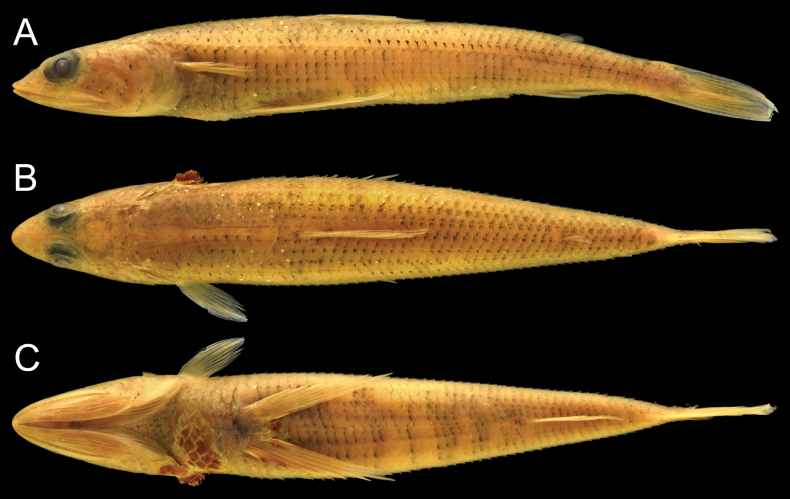
Preserved specimen of *Synodusrubromarmoratus* (AMS I. 19450-027, holotype, 73.8 mm SL, Lizard Island, Australia). **A.** Lateral view; **B.** Dorsal view; **C.** Ventral view.

*Synodusautumnus* sp. nov. is distinguished from all other valid and/or nominal species of *Synodus* by having the following characteristics: 49–51 lateral-line scales, 3.5 scale rows above lateral line, 0–5 peritoneal spots, pectoral fins long, tips extending to P–D line, posterior part of preopercle scaled, anterior palatine teeth in a discrete group, longer than posterior palatine teeth, ANF long and broad, body with 5 saddle-like blotches, and body surface below lateral line with a row of brown blotches (Russell and Cressey 1979; Cressey 1981; Waples and Randall 1988; Russell 1999, 2002; Chen et al. 2007; Prokofiev 2008; Randall and Pyle 2008; Randall 2009; Ho et al. 2010, 2016; Frable et al. 2013; Allen et al. 2017; Polanco and Acero 2021; Furuhashi and Motomura 2025; this study). In the Indo-Pacific region, the species is similar to *S.binotatus* and *S.rubromarmoratus*, but is distinguished from both by the number of lateral-line scales (49–51 vs. 52–56 in *S.binotatus*, 53–55 in *S.rubromarmoratus*) (Russell and Cressey 1979; Cressey 1981; Waples and Randall 1988; this study). In addition, the new species is distinguished from *S.binotatus* by the blotches below the lateral line forming a straight line (zigzagged in *S.binotatus*) when fresh, and from *S.rubromarmoratus* in having the anterior palatine teeth longer than those posteriorly (anterior and posterior palatine teeth same length in *S.rubromarmoratus*), teeth on the tongue numbering 29–50 (14–25), and peritoneal spots 0–5 (11–12) (Russell and Cressey 1979; Cressey 1981; this study). On the other hand, the original description of *S.rubromarmoratus* indicates a similar color pattern (color information available only as descriptive text and from black and white sketches; no color photographs known) to *S.autumnus*, the only coloration difference between the two species being the presence of eight greyish diamond-shaped markings on the body of preserved specimens of *S.rubromarmoratus* (no markings in *S.autumnus*) (Russell and Cressey 1979). However, the type specimens of *S.rubromarmoratus* lacked such markings, although they may have been lost following preservation (Fig. 6).

**Figure 7.**
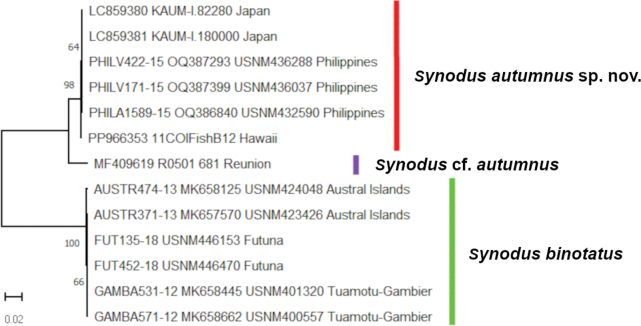
Neighbor-joining phylogenetic tree of *Synodusautumnus* sp. nov., *S.binotatus* and S.cf.autumnus based on COI sequences (598 bp). Support values (≥50 ML bootstrap probability) indicated along branches.

Only *S.autumnus* and *S.binotatus* could be compared genetically as sequences from true *S.rubromarmoratus* were not available. A 20.2% distance between the former in the COI region (598 bp) confirmed their genetic distinction (Fig. 7). In addition, a Hawaiian sequence (PP966353) was almost identical to those of *S.autumnus* from Japan and the Philippines (only 0.17% distance), and despite the former specimen’s identity not being confirmed, the very high concordance rate led to the conclusion that it was conspecific with the latter. Accordingly, the Hawaiian Islands were included in the distribution of *S.autumnus*. On the other hand, the sequence from the western Indian Ocean territory of Réunion (MF409619) differed from the above sequences by c. 4%, a value considered to represent a separate species for many fishes (Ward et al. 2005). Because a voucher specimen for that sequence could not be confirmed, Réunion was excluded from the distribution of *S.autumnus*, and the sequence is considered here as Synoduscf.autumnus. Future examination of specimens from Réunion is necessary to determine their specific identity. Although genetic information for true *S.rubromarmoratus* is so far lacking, that species is likely to form an independent clade due to its unique morphology.

### ﻿Re-assessments of previous records

*Synodusautumnus* sp. nov. is likely to have been previously recorded as *S.rubromarmoratus* from Grand Comoro Island (Heemstra et al. 2006), the Maldives (Anderson et al. 1998; Kuiter and Debelius 2006), Hong Kong (Sadovy and Cornish 2000), the Philippines (Allen and Erdmann 2012), Indonesia (Kuiter and Tonozuka 2001; Kuiter and Debelius 2006), Japan (Randall 1998; Senou et al. 2006a, 2006b, 2007), Australia (Randall et al. 1990), and the Hawaiian Islands (Randall 1998, 2005, 2007), all based on underwater photographs. However, since differentiation of the new species from *S.rubromarmoratus* requires examination of teeth and lateral-line scales, in addition to peritoneal spot numbers (see above), such photographic records cannot reliably be used for species determination. Incidentally, the photograph from Grand Comoro Island is neither of the above two species and resembles Synoduscf.randalli. This is because the lower saddle-like blotches are not reddish in *S.autumnus* and *S.rubromarmoratus* (uniformly reddish in *S.randalli* Cressey, 1981) (Cressey 1981; this study). Catalog records (e.g., Hutchins 2001; Allen and Adrim 2003; Myers and Donaldson 2003; Fricke et al. 2011) are similarly problematic; without examination of voucher specimens, they do not reflect reliable distribution records of either species.

On the other hand, specimen-based records attributed to *S.rubromarmoratus* are known from Taiwan (Cressey 1981), the Philippines (Cressey 1981), Indonesia (Gloerfelt-Tarp and Kailola 2022), and the east coast of Australia (Russell and Cressey 1979). However, examination of the specimens from Taiwan (USNM 217615, 4 specimens) and the Philippines (USNM 136282, 2) showed them to be misidentifications of *Synoduslobeli*, the specimens having 25–28 anterior gill rakers, 5.8–7.0% orbit diameter of SL and 1.6–2.1% interorbital width of SL (24–29, 5.3–7.5 and 1.8–2.1, respectively, in *S.lobeli* vs. 20–26, 6.6–7.8 and 2.0–2.7, respectively, in *S.rubromarmoratus*) (this study). A specimen (USNM 264327) from Indonesia was not examined during the present study; however, it cannot be regarded as reliable since no basis for its identification was provided in the description by Gloerfelt-Tarp and Kailola (2022). The record of *S.rubromarmoratus* from South African (Russell 2022) was accompanied only by color illustrations (pl. 33), and could not be identified in this study. Consequently, reliable records of true *S.rubromarmoratus* are available only from the east coast of Australia, where that species may possibly be endemic. This study emphasizes the need for identification of species of *Synodus* based on specimens wherever possible, rather than relying solely on underwater color photographs or specimen photographs.

## Supplementary Material

XML Treatment for Synodus autumnus

## ﻿Additional information

### Author ORCIDs

Ryusei Furuhashi https://orcid.org/0000-0001-8313-1775

Hiroyuki Motomura https://orcid.org/0000-0002-7448-2482

### Data availability

All of the data that support the findings of this study are available in the main text.
